# Pruritus Vulvae in Diabetes

**Published:** 1901-03

**Authors:** 


### Pruritus Vulvae in Diabetes.

℞ Acid. boric...................... ℥ i.+ʒ ivss.

Sodii biborat.................... ʒ i.+gr. xv.

Aq. destil....................... O ij.

M. S. Use as lotion.

—Lutaud.

